# Local Spatial Analysis and Dynamic Simulation of Childhood Obesity and Neighbourhood Walkability in a Major Canadian City

**DOI:** 10.3934/publichealth.2015.4.616

**Published:** 2015-09-08

**Authors:** Rizwan Shahid, Stefania Bertazzon

**Affiliations:** 1 Health Services Research & Evaluation, Alberta Health Services, 2430 Southport Atrium, 10101 Southport Road SW, Calgary, AB, Canada, T2W 3N2; 2 Department of Geography, University of Calgary, 2500 University Drive NW, Calgary, AB, Canada, T2N 1N4

**Keywords:** child obesity, walkability, geographically weighted regression, simulation modeling, obesogenic environment, Canada

## Abstract

Body weight is an important indicator of current and future health and it is even more critical in children, who are tomorrow's adults. This paper analyzes the relationship between childhood obesity and neighbourhood walkability in Calgary, Canada. A multivariate analytical framework recognizes that childhood obesity is also associated with many factors, including socioeconomic status, foodscapes, and environmental factors, as well as less measurable factors, such as individual preferences, that could not be included in this analysis. In contrast with more conventional global analysis, this research employs localized analysis and assesses need-based interventions. The *one-size-fit-all* strategy may not effectively control obesity rates, since each neighbourhood has unique characteristics that need to be addressed individually. This paper presents an innovative framework combining local analysis with simulation modeling to analyze childhood obesity. Spatial models generally do not deal with simulation over time, making it cumbersome for health planners and policy makers to effectively design and implement interventions and to quantify their impact over time. This research fills this gap by integrating geographically weighted regression (GWR), which identifies vulnerable neighbourhoods and critical factors for childhood obesity, with simulation modeling, which evaluates the impact of the suggested interventions on the targeted neighbourhoods. Neighbourhood walkability was chosen as a potential target for localized interventions, owing to the crucial role of walking in developing a healthy lifestyle, as well as because increasing walkability is relatively more feasible and less expensive then modifying other factors, such as income. Simulation results suggest that local walkability interventions can achieve measurable declines in childhood obesity rates. The results are encouraging, as improvements are likely to compound over time. The results demonstrate that the integration of GWR and simulation modeling is effective, and the proposed framework can assist in designing local interventions to control and prevent childhood obesity.

## 1. Introduction

Obesity is not a recent phenomenon in human history [1], yet only a few decades ago it was considered a minor problem, until its rapid and unexpected increase in the 1980s [2]. The World Health Organization (WHO) recognized obesity as a major health problem in 1997 [3], whereas in Canada, obesity as a problem was recognized as early as 1953, when the first Canadian weight-height survey was conducted [4]. Recently, 60.1% of Canadian men and 44.2% of women reported to be overweight or obese [5]. While some literature contests the existence of an ‘obesity problem’ [6], the medical literature has reported a number of health consequences associated with increased levels of body fat [7], and current obesity rates may be one of the most critical health threats facing Canadians and Americans [8]. Among American non-smokers at age 40, obesity could result in 7.1 year reduction in life expectancy for women and 5.8 years for men, while overweight could reduce life expectancy both in males and females by over 3 years [9]. In addition, the costs associated with obesity and related illnesses are estimated in $40 billion annually for the Canadian Health System alone [10]. Recent figures suggest that the rate of increase of obesity is declining in the western world, yet, if it may be reaching a plateau in this part of the world, data suggest that it is increasing at faster rates in the developing world [11].

What is even more disconcerting, child obesity has been increasing over the same decades [12]. In Canada, one in three children is classified as obese or overweight [13]. Between 1978 and 2004, child obesity increased from 3% to 8%, and overweight from 12% to 18%. There was no difference found between Canadian boys and girls aged 11 and less from 1978 to 2004 [14]. In Alberta, 26% of children are overweight or obese [15]. Childhood obesity is associated with comorbidities, such as emotional, learning, and musculoskeletal disorders in childhood, as well as early onset of type 2 diabetes and cardiovascular disease. For the first time in recent history, the youngest generation is expected to live shorter lives than their parents, because of obesity [16]. Childhood is a critical time for health and lifestyle development, and reducing childhood obesity and overweight can impact health in later stages of life, potentially reducing overall obesity prevalence. The prevention of childhood obesity represents an important opportunity to reduce the future burden of non-communicable diseases and improve the health of children and their families [17].

The World Health Organization (WHO) standard for measurement of obesity is Body Mass Index (BMI) [18]. BMI is calculated by weight in kilograms divided by the height in meters squared. In adults, BMI lower than 18.5 is considered low weight. Normal body weight ranges from 18.5 to 24.9. Overweight is defined as BMI between 25.0 and 29.9, and obese as BMI of 30 or >30. In children, age and gender specific BMI percentiles are estimated using the CDC growth charts [19]. Overweight is usually defined as a BMI between the 85^th^ and the 95^th^ percentile of children of the same age and sex; obesity is defined as a BMI at or above the 95^th^ percentile of the same age and sex. Same threshold for overweight and obesity in children was used in this research.

Obesity results from imbalance between energy intake and expenditure. The rise in obesity over the span of three to four decades has emerged more from a complex mix of social and environmental factors than from individual factors, as this time span is too short for any significant change in human genetics [12], [20]. While genetics does play a role, the environment is considered more important in the development of obesity [21]. Carnell & Wardle [22] define obesogenic environment as “the sum of the influences that the surroundings, opportunities or conditions of life have on promoting obesity in individuals and populations”. Increasing research efforts and budget allocation have been devoted to the study of obesity [23], [24]; nonetheless, some suggest that this work has yielded only limited results, in part because “social and environmental factors were not given adequate consideration within intervention design and implementation” [25]. Indeed, the distribution of obesity and overweight exhibits discernible spatial patterns within cities, as social and environmental risk factors vary over space and contribute to define local neighbourhood characteristics [26], [27]. The social and environmental factors that can promote or inhibit obesity are numerous, complex, and interconnected. Only some of them can be assessed by meaningful measures, including income level, education achievement, foodscapes [28], and neighbourhood walkability [29].

Low socio-economic status (SES) is generally associated with greater risk of overweight and obesity, both in adults and children [30]. Healthy food in North America tends to be more expensive than unhealthier options, and there is evidence that low-income families are likely to consume more unhealthy food than high-income families [31], [32]. Education impacts quality of life in multiple ways [33] and education levels are generally associated with health literacy [34]. Canadians with lower education (high school diploma or less) are at higher risk of obesity than university graduates [35]. Further, children have an increased likelihood of obesity if they live in a neighbourhood with a high percentage of people with less than high school education [36]. Among low SES individuals, recent immigrants form a vulnerable group: as they tend to be socially and economically deprived and struggle to adopt a new life style and culture, they are at risk of obesity and overweight [37]. Further, immigrants' children are at greater risk of overweight and obesity due to biosocial and cultural factors [38]. The recent Alberta Walking Survey [39] reported that Albertans in medium or high income households were much more likely to walk within their neighbourhood than those in low income households. Canadian children in low income families take approximately 1200 fewer steps per day then children in the highest income families [14]. Sports and physical activity programs provide opportunities for children to be more active on a regular basis, but such programs are expensive, and Canadian low income families are 205 times less likely to enrol their children in such programs [30].

Dietary choices are related to multiple factors, including culture, lifestyle, SES, demographics, and accessibility [40] within local food environments, or *foodscapes* [28]. Fast foods are notoriously high in energy and fat content [41], and residential proximity to fast food restaurants is associated with greater odds of obesity [23]. A recent Canadian study estimated the average increase in BMI (0.022kg/m^2^) associated with one additional fast food restaurant at the neighbourhood level [42]. Moreover, accessibility to fast food is associated with lower intakes of healthy food [43]: in Australia, children who lived within 800 m of a fast food restaurant were 38% less likely to eat fruit twice a day [12]. It is known that marketing policies, packaging, and high sugar content make fast food particularly attractive to children, with the potential consequence of establishing poor dietary habits at early ages. Further, children are mostly exposed to fast food advertisement through watching TV or other screens, hence children who are regularly engaged in unhealthy sedentary activities, are also more prone to the attraction of unhealthy foods [44].

Walking is the most frequently adopted type of regular exercise [45] by all age groups, as it does not require a high level of fitness, nor special equipment or facilities. Establishing a lifestyle that includes regular walking since an early age may be the best way to develop active and healthy lifelong habits, as childhood sports too often focus on talent scouting, nor do fitness programs focus on promoting physical activity outside the allotted time, let alone their financial implications [30]. Walking can be an elective activity, i.e., going for a walk, which takes time and competes with other choices, e.g., watching a movie or playing a game. In addition, walking is a natural mode of transportation, which can take one to school, public transit, and other destinations; as such, it is part of one's daily routine, taking no extra time, nor competing with other activities. However, any form of walking requires suitable conditions [46], which include, at a minimum, a reasonably safe and pleasant environment. In addition, transportation walking requires accessibility; i.e., destinations must lie within *walking distance* of one's residence. This is an onerous requirement, which effectively restricts access to this form of walking: an important role in one's ability to walk routinely is played by their residential neighbourhood [47]. Neighbourhood walkability is considered an important determinant of physical activity and body weight [48]–[50] and, in children, high walkability is positively associated with active park use [51], with the added benefit of increased levels of physical activity and more time spent outdoors [52]. Consistently, proximity to parks further influences physical activity. Laxer and Janssen [48] found that the risk of physical inactivity was 28% to 37% higher for youth living in neighbourhoods with low park space, compared to youth living in neighbourhoods with moderate to high park space.

Obesity and overweight may be, to a considerable extent, local phenomena, and a careful enquiry into local obesogenic environments may lead not only to a greater understanding of local-scale obesity risk factors, but potentially to more effective local interventions aimed at obesity reduction. To reduce childhood obesity, one critical task is to identify which risk factors matter, where they matter, and how obesity prevalence would be impacted if these risk factors could be modified. While local interventions necessarily yield more modest results than city- wide ones, they may also be more cost-effective and easier to implement, even in the short term. Health geographers and spatial epidemiologists have investigated obesity using a variety of research methods, including spatial analysis [26], [27] and simulation models [53], [54]. However, in order to evaluate the potential effects of local-scale interventions, temporal and spatio-temporal dynamics should be evaluated at the local scale [55]. This study presents a promising integration of spatial analysis and simulation modeling. Local spatial analysis (geographically weighted regression, or GWR) [56] is used to assess the relationship of childhood obesity and overweight with their risk factors at the neighbourhood level. Simulation modeling is then used to simulate localized interventions aimed at reducing childhood obesity, and to estimate their potential impact over time, by analyzing *what-if* scenarios [57]. The local analysis presented is multivariate, so that it can assess the relative contribution of a set of measured factors. Yet, the focus is on neighbourhood walkability. Of the many factors associated with childhood obesity, neighbourhood walkability is important for the development of healthy habits at early ages; it can impact children more than adults, by promoting park and playground use, hence more physical activity and more outdoors time. Further, compared to other factors, e. g., SES, interventions on neighbourhood walkability are relatively simple, inexpensive, and can have a measurable impact over a relatively short time frame.

Local spatial analysis identifies target neighbourhoods where a hypothetical intervention has the greatest probability of success, that is, neighbourhoods where walkability is low, and the prevalence of obese children is high and significantly associated with walkability. An intervention consisting of increasing neighbourhood walkability is simulated only on the subset of neighbourhoods with the specified characteristics. Spatial and dynamic models then assess the effect of the simulated intervention on each neighbourhood after a specified period. A second spatial analysis assesses the association of obesity and walkability after the intervention. The results show that increased walkability reduces child obesity prevalence. It also reduces the significance of walkability as a factor of obesity, thereby exposing other critical factors that could be targeted by further interventions.

## 2. Study Area and data

Calgary is a major Canadian city, whose current urban area extends over a wide land area (approximately 825 km), with sprawling suburbs, largely car dependent, and dominated by single-family dwellings. In 2006, Calgary had the second largest average commute distance in Canada [58].The city largely grew in the automobile era, and neighbourhoods built before the 1950s are more walkable than more recent ones. As of the Census of Canada survey [59], the population of Calgary was 988,193 spread over 185 residential neighbourhoods. These neighbourhoods were chosen as the primary unit of analysis because they encompass a sense of identity; it has long been recognized that people choose to live in certain neighbourhoods and feel association with the communities they live in [60], [61]; further, children usually play close to where they live [62]. The average population of residential neighbourhoods is 5,341 residents, and 1,320 of them are children (age 0 to 19).

### 2.1 Children BMI

The province of Alberta (Canada) recommends that children receive a set of vaccinations (diphtheria, tetanus, acellular pertussis, polio, measles, mumps, rubella, varicella, pneumococcal conjugate), and families are invited to have their children vaccinated at age 4.5: most children are vaccinated before the end of grade one [63]. At the time of the vaccination, clinics collect other information on each child, including their BMI and residential address. The province maintains a database (PHANTIM - Primary Health Activity Network Timely Information Management) containing information on each child who received the vaccination at a public health clinic. For this study we obtained data on residence location (postal code), and BMI percentile from 2005 to 2008. The PHANTIM data covers more than 80% of the Calgary child population of this age group [34].

**Figure 1.**
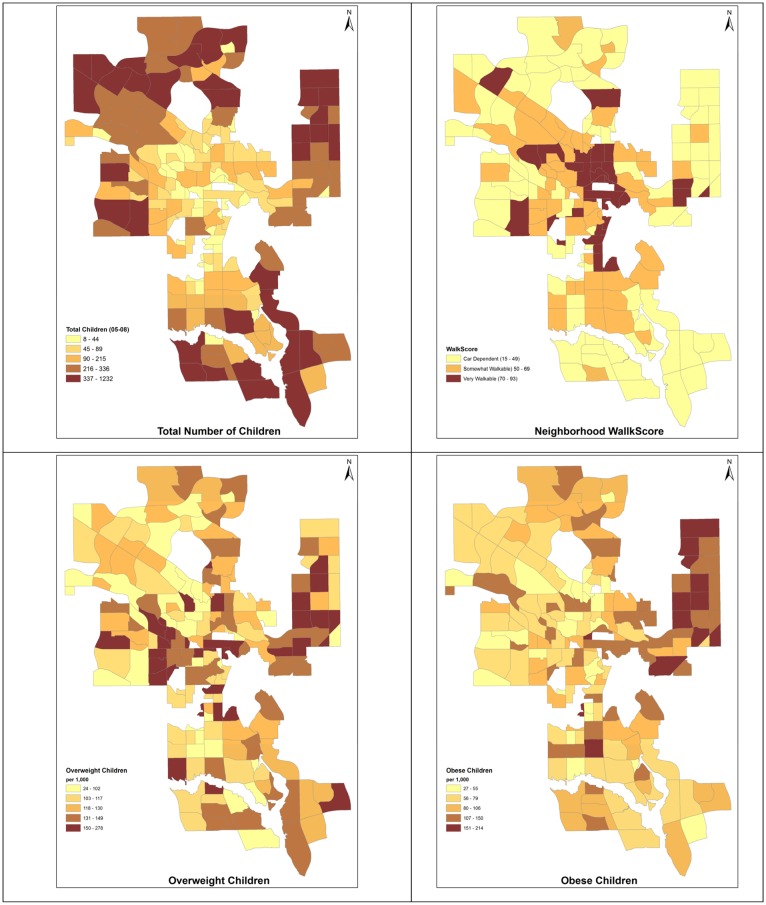
Spatial distribution of children, walkscore, overweight children and obese children.

The residential postal code of each child was geo-coded using Alberta Health Postal Code Translator File [64] and aggregated at the neighbourhood level, using ESRI (Environmental System Research Institute) ArcGIS software, [65]. Of the 185 neighbourhood, 11 were excluded from the analysis, due to low population or missing variables, resulting in 174 valid units. Upon combining the 4 years of PHANTIM data for age 4.5 to 6 years old children, the total sample size was 37,460 children, with an average of 215 children per neighbourhood. Of these children, 3,626 (9.68%) were obese, 4,669 (12.46%) overweight, 27,397 (73.14%) healthy weight, and 1,768 (4.72%) underweight. Given their relatively low proportion, and the focus of this study, underweight children were not included in the analytical models. Alternatively, under-weight children could have been merged with healthy weight children, but that may have inaccurately inflated the proportion of healthy-weight children. For the analyzed age group, the average number of obese children per neighbourhood is 27, ranging across neighbourhoods from 2.7% to 21.4%; the average number of overweight children is 21, ranging across neighbourhoods from 2.4% to 27.8%. Prior to using it in regression analysis, this variable was standardized, as suggested by Chalkias et al. [26] and Preston et al. [66], by taking the proportion of overweight and obese children to total children and multiplying by 1000. The transformed overweight and obese variables are referred to as ‘normalized overweight’ and ‘normalized obese’ (see Figure 1).

### 2.2 Census Data

Data on socioeconomic status, including income, education, and immigrant population for each neighbourhood were obtained from Statistics Canada for the 2006 Census [59]. Income is represented by the census variable “median family census income”, which provides the median income of each neighbourhood, and refers to the family income across all sources, including employment income, income from government programs, pension income, investment income and any other income [67]. Annual income values were converted to $1000s. Education is represented by the census variable “people with no certificate, diploma or degree”, which amounts to the portion of the population aged 25 and older who did not complete high school. Immigrant population is represented by the census variable “immigrants”, which refers to those who have been granted the right to live in Canada permanently by immigration authorities. Some immigrants have resided in Canada for a number of years and other landed recently [67]. The education and immigrant population variables were normalized by dividing them by the total child population in each neighbourhood and multiplying by 1000 [26], [66].

### 2.3 Proximity to Fast Food Restaurants

Walking or driving access to fast food restaurants is considered an indicator of the consumption of fast food [12]. In the absence of data on average fast food consumption in each neighbourhood or number of visits to fast food outlets by families, proximity is used as an indicator of fast food consumption in each neighbourhood. Here, proximity is defined as the number of children living in the walkshed, or walkable distance of fast food outlets. Standard Industrial Classification (SIC) codes were used to select eating places. SIC codes were established in 1987 by the US Government to group businesses and to identify primary activities [68]. In the City of Calgary, 61% of neighbourhoods have at least one fast food restaurant, and the number of fast food restaurants in each neighbourhood ranges between 0 and 24. A generally accepted threshold for the walkshed is 800 m [12], [34]. Therefore, a buffer of 800 meters around each fast food restaurant was generated based on the DMTI road network (licenced to the University of Calgary) using ESRI Network Analyst. Postal codes within the buffer were selected. Children, from the PHANTIM dataset, living in the selected postal codes, were aggregated and normalized.

### 2.4 Proximity to Parks

Proximity to parks is an indicator of neighbourhoods that promote childhood physical activity in children [48]. The focus of this study was on local parks, which can be accessed by walking on a regular basis, for example on the way home from school. In contrast, large parks serve a population greater than the neighbourhood residents, and tend to be accessed by car and mostly on weekends [34]. For this reason, parks greater than one square km were removed from the analysis. Proximity to local parks was assessed with the 800 m threshold.

### 2.5 Walkability

Neighbourhood walkability is a complex property, determined by several features, and difficult to measure [69]. For practical reasons, the Walkscore™ index was used in this study. Walkscore™ is a free-to-use, publically available index of neighbourhood walkability developed by Front Seat Management Company [45]. It calculates the walkscore of addresses as a weighted distance to local amenities. These amenities are divided into five categories: education, recreational, food, retail, and entertainment. The index calculates straight-line distance to the closest facilities in each category. These distances are summed and weighted by a distance decay function applied equally to each facility category [70]. The result is then normalized to an index ranging from 0 to 100, where 0 is not walkable, or car dependent, and 100 is the most walkable [49].

Walkscore values for each residential community were downloaded from the Walkscore web site [71]. Based on this index, only 33 of 174 Calgary neighbourhoods are highly walkable (19%), 57 are somewhat walkable (33%) and 84 are car dependent (48%). Nearly 65% of children live in car dependent communities, 26% in somewhat walkable and only 9% in highly walkable neighbourhoods (see Figure 1). While census data where drawn from the 2006 survey, in accordance with the clinical records, walkscore data were only available for 2012. However, walkscore typically does not vary over a short span of time. The location of amenities tends to remain the same in and around the neighbourhoods; moreover, the distance weighting method helps to represent the stability of the neighbourhood walkability.

### 2.6 Pathway Length

Calgary has the most extensive urban pathway network in North America [72], connecting its parks, natural areas, and part of its neighbourhoods. Pathways provide dedicated routes for biking, rollerblading, walking, running, etc. Since the walkscore index is not calculated on pathways, this variable was included to provide additional information on walking opportunities in each residential neighbourhood. Pathway data were downloaded from the City of Calgary website and their length was measured (in Km), for each neighbourhood, in ArcGIS.

## 3. Methods

Child overweight and obesity exhibit distinct spatial patterns across the city (Figure 1). Therefore, two distinct spatial models analyze their association with each risk factor. Further, healthy-weight children are considered at risk of becoming overweight, and overweight children are considered at risk of becoming obese in the simulation model [9]. Correlation and cross- correlation analysis was performed between the dependent and independent variables at the global level. The analysis suggested only some moderate multicollinearity (presence of highly inter-correlated independent variables). This issue should not be problematic for local level analysis. Tests of multicollinearity in local analysis are relatively recent and there is currently little consensus about their reliability in the literature [73]–[75].

### 3.1 Geographically Weighted Regression

Geographically Weighted Regression (GWR) is a local spatial analytical technique, which extends the traditional global regression framework (1) by estimating local parameters for a geographic window moving across a dataset [56].

$$Y_{i}=\beta_{0}+\beta_{k}X_{ik}+\varepsilon_{i}$$ (1)

The typical GWR model is expressed by (2).

$$Y_{i}=\beta_{0}\left(u_{i},v_{i}\right)+\sum_{k}\beta_{\text{k}}\left(ui,vi\right)X_{ik}+\varepsilon_{i}$$ (2)

Besides standard regression notation, the term *(u_i_, v_i_)* denotes the geographic coordinates of the *i^th^* point in space. Hence, equation (2) yields *i* local regressions over each sample point, and a total of i*k regression coefficients. If the relationships do not exhibit spatial variability, (2) coincides with (1).Under standard regression assumptions, both equations can be solved by the ordinary least squares (OLS) method, which includes a spatial weight for (2). OLS regression coefficient estimates for the GWR model are expressed by (3):

$$\hat{\beta }\left(u_{i},v_{i}\right)={\left(X^{T}W\left(u_{i},v_{i}\right)X\right)}^{-1}X^{T}W\left(u_{i},v_{i}\right)Y$$ (3)

Where ${\hat{\beta }}_{k}\left(u_{i},v_{i}\right)$ represents an estimate of β_k_(u_i_, v_i_), and W(u_i_, v_i_) is a spatial weights matrix. GWR assumes that observations near each point *i* exert more influence in the estimation of parameters at location *i* than those further away [56]. Several weighting functions are used to define local areas^1^ where GWR parameters are estimated. Among the most common are fixed and adaptive kernels, where kernels are drawn around each sample point. With fixed kernels, a buffer is defined by a fixed distance, and local kernels may contain a varying number of observations, if the latter are not uniformly distributed. With adaptive kernels, the distance can vary, in order to retain a constant number of nearest neighbours in each kernel. In this study, an adaptive kernel was preferred, as spatial units (urban neighbourhoods) vary in size and shape over the study area. For adaptive kernels, bandwidth is defined as the number of nearest neighbors forming each local area. The weight inside the bandwidth reaches monotonically zero as the distance increases [76]. Akaike Information Criterion (AIC) is generally used to select the optimal bandwidth [77]. In this application, a bandwidth of 50 nearest neighbours was used in both regressions.

GWR yields a large number of estimates, which are not easily tabulated and are generally mapped to visualize their spatial variation [78]. Mapping the statistical significance (measured by the t value), alongside each parameter, provides further insights on the association between dependent and independent variables. In mapping the t-values, we categorized them according to the common significance thresholds of 90%, 95% and 99% [78]. Still, these process typically results in numerous maps (k*2) for multivariate regressions. In this paper, we introduce a novel mapping method that combines in single map information about individual parameter and their significance, along with the portion of variation explained by each local model.

### 3.2 Simulation Modeling

Simulation modeling methods are used to understand dynamic complex systems and to predict the outcome of change [79]. They include system dynamics, which is used to study the relationship between dynamic behaviour and structure in terms of feedback loop mechanisms [80]. In a system dynamics model, the phenomenon under study is conceptualized as a series of connected stock and flow diagrams. Behind each stock and flow, there is a set of mathematical equations that determine how stocks and flows are connected and interact with each other. Such models can be run in a steady state and change (intervention) can be simulated based on different input scenarios [81]. In short, a system dynamic model provides a simulation environment to learn how action in the present can trigger plausible reactions over time [82].

**Figure 2.**
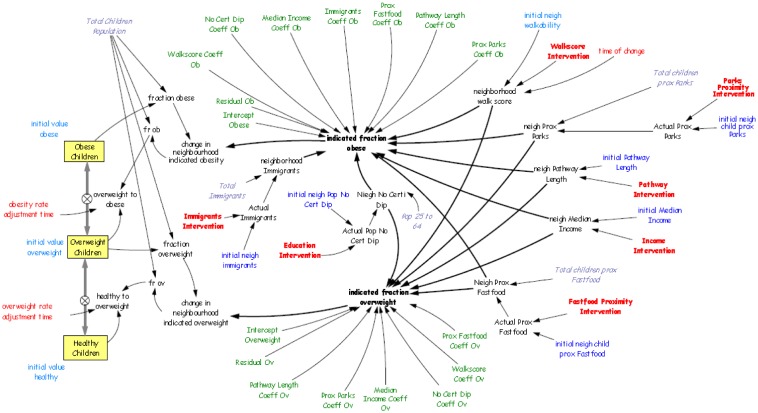
Complete Simulation Model.

In this study a simulation system dynamics model was specified in Vensim™ [83]. The simulation model was structured around the GWR obesity model. Three weight categories (healthy-weight, overweight and obese) were included in the model as stocks, representing the total number of children in each category. The stocks were connected with flow arrows representing potential transitions of children from one stock to the other, defining the variables ‘*overweight to obese*’ and ‘*healthy to overweight*’. These variables governed the flow rate, which was directly or indirectly influenced by interventions. In order to represent each neighbourhood separately, 174 simulation models were defined using subscripts with each stock and variable. The model was constructed as progressive: each child enters the system in any of the stocks based on their BMI percentile, and can only move to the adjacent stock; i.e., it is not possible to move directly from obese to healthy-weight and vice versa. For simplicity, one year was defined as time it takes to perform walkability intervention and for the obesity rate adjustment time. Figure 2 shows the complete simulation model with all the GWR coefficients and variables.

### 3.3 Integration and simulation

Once the simulation model was tested in equilibrium state, interventions were introduced. The GWR model results were inspected to identify neighbourhoods where childhood obesity is high, and walkability is low and significantly associated with childhood obesity. A local walkability intervention was simulated on these neighbourhoods, based on two alternative scenarios: a 10-point, and a 20-point increase in walkscore. While all variables are modifiable at some scale, no other variable was altered. Based on 2005–2008 child data, the model ran a simulation from 2009 to 2015. After this initial intervention, GWR models were re-run on the new walkscore values, yielding a smaller subset of neighbourhood where walkscore was still significantly associated with obesity.

**Figure 3.**
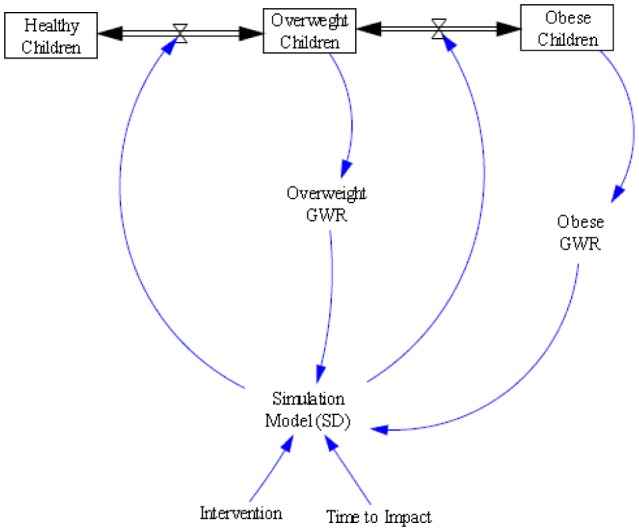
Integration of GWR and SD Simulation Model.

The concept diagram of the integration of GWR and simulation model with the feedback process is shown in Figure 3. The process was repeated for the 2 scenarios. The results of the GWR models before and after the intervention were compared for each targeted neighborhood, to assess the impact of the intervention on obesity prevalence and the new significance of the association between walkscore and obesity.

## 4. Results

Descriptive statistics for all variables are summarized in Table 1. The table shows the global statistics (for the entire study area). As show in the Table (Standard Deviation), the variables display large variability across neighbourhoods. Summaries for two sample communities, Forest Heights (FHT) and Saddle Ridge (SAD) are also shown, to exemplify how data vary across neighbourhoods.

**Table 1.** Descriptive statistics of dependent and independent variables.

*174 neighbourhoods*	Min.	Max.	Mean	Median	Std.Dev.	Forest Heights	Saddle Ridge
Total children	8	1232	215	158	215.02	212	702
Overweight children	1	139	27	18	26.55	31	79
Obese children	1	122	21	12	23.43	27	108
Normalized overweight children	24	278	128	124	38.08	146	112
Normalized obese children	27	214	92	86	38.34	127	154
Immigrant population	93	818	231	208	97.48	368	469
Education	0	381	102	77	77.85	289	167
Median census family income	27	280	96	88	36.90	58	74
Proximity to fast food restaurants	0	1000	388	303	340.37	561	153
Proximity to parks	0	1000	845	942	204.41	1000	523
Walkscore	15	93	52	50	17.68	72	43
Pathway length	0	22	4	3	4.04	1	4

### 4.1 Spatial Analysis

The results of the obesity GWR are summarized in Table 2, where summary results are presented in the table, while the local regression results are visualized in detail in the map.

**Table 2.** Geographically weighted regression of child obesity prevalence.

	**Regression Summary**							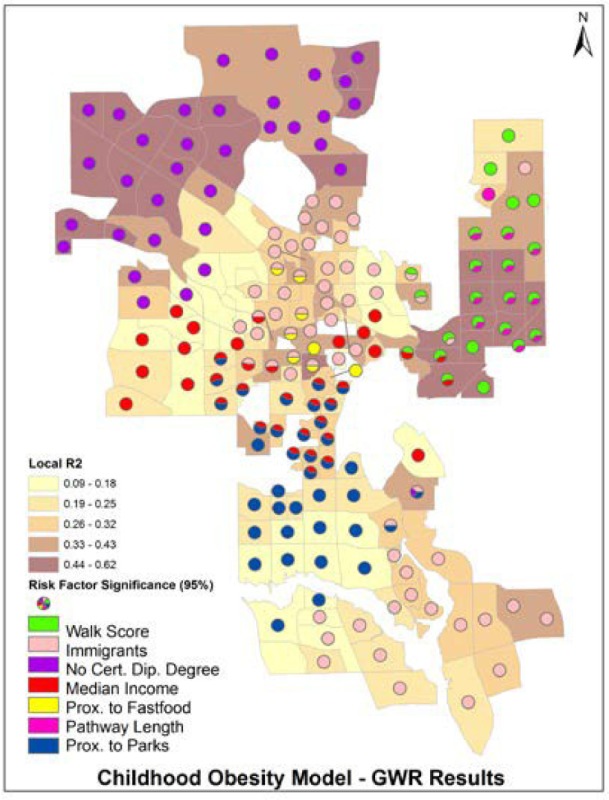
	**Regressions**	174	**Local Sample**		50			

	**Mean Local R2**	0.30						
	Local R^2^ Min.	0.09	Local R^2^ Max.		0.62			

	**Coefficients**	**Min**	**Max**		**Mean**	**Range**		
	Education	-0.31	0.39		0.06	0.70		
	Immigrants	0.00	0.34		0.08	0.34		
	Family income	-1.04	0.27		-0.12	1.31		
	Prox. Fast food	-0.03	0.04		0.00	0.07		
	Prox. parks	-0.09	0.04		-0.02	0.13		
	Walkscore	-1.59	0.77		-0.05	2.36		
	Pathway length	-4.98	2.92		-1.26	7.90		

	**Obese Children**							
	Total	3,626		Percent	9.7			
	Average	21		St. Dev	23.4			


The negative association between obesity and walkability is significant (95%) in 24 communities. As shown in Table 2, all of these neighbourhoods are located in the Northeast, an area where childhood obesity is mostly above the city average, ranging from 64 to 192 per 1,000 children, whereas the city average is 92 per 1,000 children. Walkability in those communities is generally low, as 63% are car dependent (compared to 48% over the whole city) 25% somewhat walkable (vs. 33% over the whole city), and 12% highly walkable (vs. 19% over the whole city).

### 4.2 Simulation

Two walkscore scenarios (10-point increase and 20-point increase), were computed for the 24 identified neighbourhoods. Both scenarios yielded a reduction in the obese children stock in all 24 neighborhoods.

**Figure 4.**
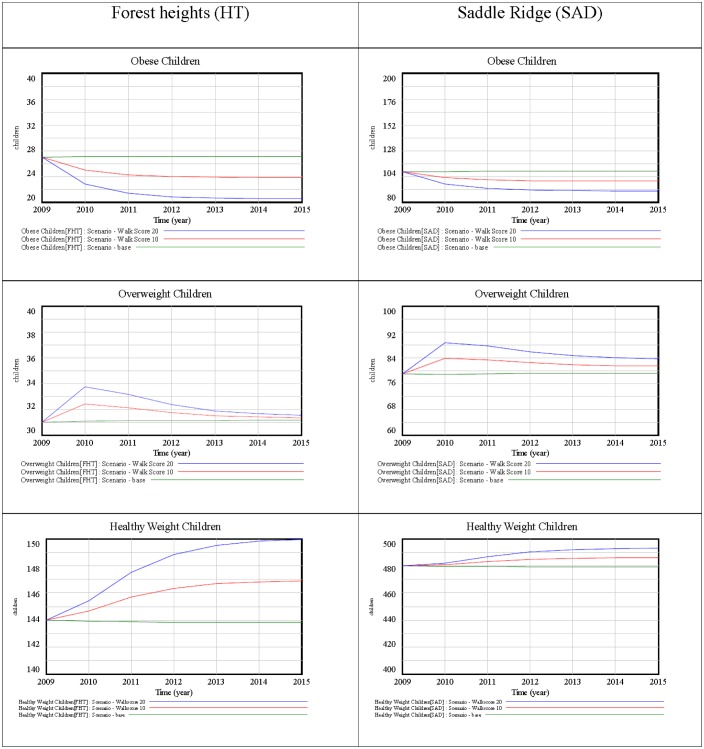
Simulation results of two selected neighbourhoods.

Figure 4 exemplifies the simulation results over the 6-year period. Due to the large number of neighborhoods, the results are shown in detailed only for two sample communities, as in Table 1, above. For each neighborhood, the simulations show: Scenario: “base” (no intervention); Scenario: “Walkscore10” (10 point increase); and Scenario: “Walkscore20” (20 point increase). The plots in Figure 4 show the impact of each simulation on the three stocks of children: healthy-weight, overweight, and obese.

“Walkscore10” decreased the number of obese children, triggering a flow from the obese to the overweight stock. However, walkability was not significantly associated with overweight in these two sample communities. While the parameter values were small, the flow between the overweight and the healthy stock was lower than the flow between obese and overweight stocks. This backup phenomenon resulted in an influx of overweight children during the first year, as a number of children transitioned from the obese to overweight stock, but this transition was not followed by a proportional transition from the overweight to the healthy-weight stock (due to the non-significant association between overweight and walkscore). Nonetheless, a modest increase in the number of healthy-weight children is observed in both neighborhoods. “Walkscore20” followed the same pattern as “Walkscore10” but it was more effective. “Walkscore20” further decreases the obese children, created higher influx of overweight children and increased the number of healthy children.

Both simulations output new numbers of children in each stock, in each neighborhood. The number of obese children was normalized and fed into the GWR obesity prevalence model, which was run again to model the new obesity prevalence (Figure 3). Table 3 shows the comparison of the baseline parameter values with the parameters of “Walkscore10” and of “Walkscore20” in the 24 targeted neighborhoods.

Both “Walkscore10” and “Walkscore20” yielded increased numbers of healthy-weight children and decreased number of obese children. Most neighbourhoods exhibit the backup phenomenon of increased overweight children; however, this is only due to the transition from obese to overweight, as in no case has the number of healthy-weight children decreased, or the number of obese children increased. Both simulations decrease the significance of walkability as a risk factor of obesity: with “Walkscore10” it falls below 90% in three neighbourhoods (highlighted in light grey), from 95% to 90% in one neighbourhood (bold), and from 99% to 95% in two neighbourhood (highlighted in dark grey); with “Walkscore20” it falls below 90% in five neighbourhoods, from 95% to 90% in one neighbourhood, and from 99% to 95% in four neighbourhoods.

**Table 3.**
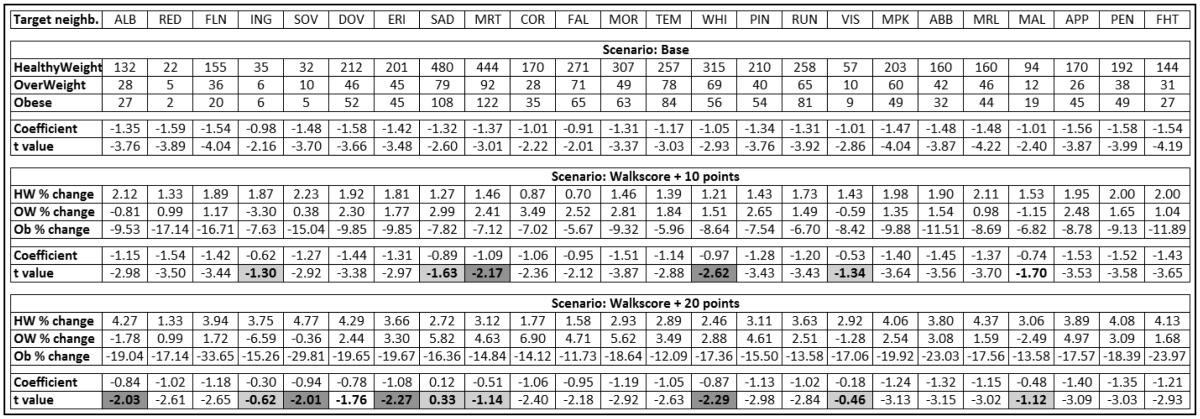
GWR results for “Base”, “Walkscore10”, and “Walkscore20”

## 5. Discussion

Neighbourhood walkability is desirable and generally associated with higher level of physical activity, yet in Calgary walkability tends to be higher in areas where the presence of children is lower (Figure 1). This measured relationship is likely an indication of other underlying relationships, such as that between walkability and SES, and between walkability and distance from the city core; these latent relationships may also compound and confound the measured relationship between walkability and childhood overweight and obesity. In our multivariate analysis of childhood obesity, the measured factors are simultaneously entered in each local regression. The model suggests that poor walkability alone is not necessarily associated with higher rates of childhood obesity; rather, it identifies a small set of deprived neighborhoods, where poor walkability, along with high density of children, lower socio- economic status, lower education attainments, and recent immigration are associated with higher childhood obesity rates (Table 2).

The simulated increase in walkability in the neighbourhoods identified by the obesity model had the two-fold effect of reducing child obesity rates as well as decreasing the significance of walkability as a risk factor. The decline in the number of obese children was accompanied by an increase in the number of overweight children, due to the influx of children transitioning from the obese stock. The overall result was a modest, but measurable increase in the number of healthy-weight children, which was noticeable after only one-year interval. Though it may take more time for the impact to be fully realized, this results is encouraging, as the improvement will likely compound over time. Addressing the backup phenomenon of overweight children requires more research in the relationship between walkability and overweight in those communities. The decreased significance of the association between childhood obesity and walkability appears as a positive result, which, on one hand, may indicate that obesity rates have become ‘normal’ in those neighbourhoods; on the other hand, it exposes other factors within the multivariate model, that can be targeted by subsequent interventions. For example, ‘Immigrant population’, ‘Median census family income’ and ‘Pathway length’ are significantly associated with overweight in the targeted neighborhoods. Interventions on pathway length maybe feasible, and lead to improved neighborhoods accessibility, resulting in greater rates of active commute (walking or cycling), in access to healthier food options and, finally, yielding measurable reductions not only of the obese but also of the overweight child population.

### 5.1 Walkscore index

The Walkscore index is an imperfect measure of neighbourhood walkability. It is calculated for a generic population, and includes amenities that are not relevant to children [84]. Indeed, the majority of the literature on walkability indices focuses on adults [45], [49] and the few studies that focus on children [69], [85] are applied to small areas or school districts, therefore they cannot be immediately transferred to city neighbourhoods. Walkscore™ does not account for weather, neighbourhood safety and crime rates that may prevent people from walking in their neighbourhoods [49]. Grigsby-Toussaint et al. [62] and Finn et al. [86] did not find significant seasonal influence on the total physical activity in preschool children; however Calgary is subject to harsh winter conditions, and with several days each winter with temperatures below −20°C and strong arctic winds, when spending more than 15 minutes outdoors is not recommended for adults, let alone children. Further, Walkscore ™ does not account for self- selection, that is, people who like to be active choose to live in active neighbourhoods that provide amenities of their interest [29]. Self-selection may generate an overestimate of the association between neighbourhood environment and physical activity.

Despite all its limitations, Walkscore remained the only practical option for this study. Weinberger and Sweet [87] deemed Walkscore a robust and transferable measure, hence a useful proxy for the walking behaviour in an area. Carr et al. [49] supported these findings, further deeming the index valid and reliable, and a reasonable proxy for neighbourhood density and access to nearby amenities. Duncan et al. [45] validated the index for estimating neighbourhood walkability and confirmed its validity in multiple geographic locations and at multiple spatial scales [49]. Due to its ease of use, constantly updated database and simple, user friendly interface, walkscore has considerable potential for public health research [70].

### 5.2 Local dynamics and neighborhood level interventions

Childhood obesity is largely a spatial phenomenon, associated with local environments and neighbourhood characteristics. Targeted interventions on at-risk neighbourhoods are likely to have lesser impact, but also lesser cost than city-wide (*one-size-fit-all*) interventions. The method presented in this paper helps to identify which interventions are suitable for which neighbourhoods and to evaluate their impact locally. Implicitly, the models identify neighbourhoods that are not in need of immediate attention. Further, the local analytical framework can aid the definition of diversified local policies, such as incremental walkability increases in one neighbourhood, and a combination of walkability and pathway length intervention in another one. Thus, the overall effect on obesity and overweight can be increased, while achieving cost reduction and improved resource allocation. All the data were sampled at the residential neighbour level, and all the analyses were conducted at the same level. In recognition of the ecological fallacy [88], as well as modifiable area unit problem [89],[90], we wish to emphasize that our analytical results are only valid at that scale, and no inference can be drawn at different scales. The scale used in this analysis involved the use of relatively small spatial units, which resulted in the removal of 11 neighbourhoods with low population numbers. While this was not ideal, the alternative would have been the use of larger spatial units. However, this would have led to less applicable analytical results, and possibly to biased results, as high population neighbourhood may dominate low population ones.

Among the risk factors of childhood obesity, walkability lends itself to relatively easy interventions. Yet, changes to developed neighbourhoods occur slowly and infrequently, and physical interventions (e. g., widening sidewalks, controlling traffic, improving safety and streetlights) require specific and potentially large budgets. An alternative to physical interventions can be the allocation of resources to the targeted neighbourhoods, in the form educational programs and awareness campaigns, with the active involvement of schools and community associations.

While studies analyzed walkability [29] and applied spatial analytical methods to model obesity [31] in Calgary and elsewhere, no other study has analyzed the geographical differences of children overweight and obesity separately, at fine spatial resolution, assessing their association with neighborhood-level risk factors. Increasing neighbourhood walkability does not imply that residents will walk more, but it can reduce one of the barriers hampering an active lifestyle for children (and adults). An important outcome of this research is an improved understanding of the complex, local environmental determinants of childhood obesity. Allocating resources only where they are needed can potentially overcome four disadvantages of planning city-wide interventions: more resources are required for city-wide interventions; local areas in greater need may not get enough resources; in some areas interventions may backfire, creating new problems; and evaluation of broad intervention may require more resources and yield misleading results due to averaging, as opposed to local-scale analysis.

## 6. Conclusion

Efforts to reduce and prevent childhood obesity can lead to overall reduction of obesity and to a healthier future, but require a thorough understanding of child obesity risk factors and the identification of vulnerable areas. Neighbourhood walkability is among the most important determinant of childhood physical activity and body weight. In this paper, overweight and obesity risk factors were analyzed separately, at the neighbourhood level. Using an innovative integrating of local analysis with simulation modeling, we identified target neighbourhoods where interventions were more likely to succeed. Target neighbourhood were identified by geographically weighted regression (GWR), which is a powerful tool to assess local relationships, yet does not address the potential outcome of *what-if* scenarios. This gap was filled by integrating GWR with simulation modeling, which allowed for a preventive evaluation of the potential success of the planned interventions in the targeted neighborhoods. The simulated intervention yielded a modest but measurable reduction in the number of obese children over a short time period. Further, it reduced the significance of walkability as a risk factor in the targeted neighbourhoods, thereby exposing other critical factors that could be targeted by subsequent interventions. The method is effective and showed promising results. It is general in nature that can be extended to the analysis of other localized public health issues.

## Footnotes

1 These areas are generally referred to as “neighbourhoods”, but we avoid using this term to avoid confusion with the urban neighbourhoods analyzed in this study.

## References

[1] Ulijaszek Sj, Lofink H (2006). Obesity in biocultural perspective. Ann Rev Anthropol.

[2] James WPT (2008). The epidemiology of obesity: the size of the problem. J Inter Med.

[3] WHO: World Health Organization (1997). Obesity: Preventing and Managing the Global Epidemic. Report of a WHO Consultation on Obesity.

[4] Pouliou T, Elliott SJ (2010). Individual and socio-environmental determinants of overweight and obesity in urban Canada. Health & Place.

[5] Statistics Canada (2011). Overweight and obese adults (Self Reported).

[6] Colls R, Evans B (2014). Making space for fat bodies?: A critical account of ‘the obesogenic environment’. Progress Human Geog.

[7] Cugnetto ML, Saab PG, Llabre MM (2008). Lifestyle factors, body mass index, and lipid profile in adolescents. J Pediatr Psycho.

[8] Catenacci VA, Hill JO, Wyatt HR (2009). The obesity epidemic. Clin Chest Med.

[9] Petit CL, Berthelot J-M (2006). Obesity-a growing issue. Health Reports.

[10] Behan DF, Cox SH [Internet]. Obesity and its relation to mortality and morbidity costs, The Society of Actuaries Report.

[11] CDC Newsroom [Internet].

[12] Fraser LK, Edwards KL (2010). The association between the geography of fast food outlets and childhood obesity rates in Leeds, UK. Health & Place.

[13] Levy E, Zambo ZM, Edellm D, Borys J-M (2015). The potential of reducing the prevalence of overweight and obese children in Canada using the EPOSE methodology. Can J Diabetes.

[14] Shields M (2006). Overweight and Obesity among children and youth. Health Reports.

[15] Alberta Health Services: Obesity Facts [Internet]. http://www.albertahealthservices.ca/7467.asp.

[16] Belluck P The New York Times [Internet]. http://query.nytimes.com/gst/fullpage.html?res=9F01E3D7133CF934A25750C0A9639C8B63&sec=health&spon=&pagewanted=all.

[17] WHO: World Health Organization (2014). Obesity: Preventing and Managing the Global Epidemic.

[18] Dehghan S, Akhtar-Danesh N, Merchant AT (2005). Childhood obesity, prevalence and prevention. Nutrition.

[19] Kuczmarski RJ, Ogden CL, Grummer-Strawn LM, Flegal KM, Guo SS, Wei R (2000). CDC growth charts: United States. Adv Data.

[20] Smith DM, Cummins S (2009). Obese cities: how our environment shaped overweight. Geogra Com.

[21] Stunkard AJ, Peña M, Bacallao J (2000). Factors in obesity: current views. Obesity and Poverty: A New Public Health Challenge. Scientific Publication 576.

[22] Carnell S, Wardle J (2007). Measuring behavioural susceptibility to obesity: validation of the child eating behaviour questionnaire. Appetite.

[23] Burgoine T, Alvandies S, Lake AA (2013). Creating ‘obesogenic realities’; do our methodological choices make a difference when measuring the food environment?. Inter J H Geog.

[24] Spence JC, Cutumisu N, Edward J (2009). Relation between local food environments and obesity among adults. BMC Public Health.

[25] Marks J, Barnett LM, Foulkes C (2013). Using social network analysis to identify key child care center staff for obesity prevention interventions: A pilot study. Obesity.

[26] Chalkias C, Papadopoulos AG, Kalogeropoulos K (2013). Geographical heterogeneity of the relationship between childhood obesity and socio-environmental status: empirical evidence from Athens, Greece. App Geog.

[27] Wen TH, Chen DR, Tsai MJ (2010). Identifying geographical variations in poverty- obesity relationships: empirical evidence from Taiwan. Geospatial H.

[28] Lake AA, Burgoine T, Stamp E, Grieve R (2012). The foodscape: classification and field validation of secondary data sources across urban/rural and socio-economic classifications in England. Inter J Behavi Nutrition Physical Act.

[29] McCormack GR, Shiell A, Giles-Corti B (2012). The association between sidewalk length and walking for different purposes in established neighborhoods. Inter J Behavi Nutrition Physical Act.

[30] Spence JC, Holt NL, Sprysak CJ (2012). Non-refundable tax Credits are an inequitable policy instrument for promoting physical activity among Canadian children. Can J P H.

[31] Shannon J (2014). Food deserts: Governing obesity in the neoliberal city. Progress Human Geog.

[32] Inagami S, Cohen DA, Brown AF, Steven MA (2009). Body mass index, neighbourhood fast food and restaurant concentration, and car ownership. J Urban Health: Bulletin of the New York Academ Med.

[33] Niedzwiedz C, Katikireddi SV, Pell JP, Mitchell R (2012). Life course socio-economic position and quality of life in adulthood: a systematic review of life course models. BMC Public Health.

[34] Potestio ML, Patel AB, Powell CD (2009). Is there an association between spatial access to parks/green space and childhood overweight/obesity in Calgary, Canada?. Inter J Behavi Nutrition and Physical Act.

[35] Tjepkema M (2006). Adult obesity. Health Reports.

[36] Janssen I, Boyce W, Simpson K, Pickett W (2006). Influence of individual- and area-level measures of socioeconomic status on obesity, unhealthy eating, and physical inactivity in Canadian adolescents. Am J Clinic Nutrition.

[37] Belanger-Ducharme F, Tremblay A (2005). Prevalence of obesity in Canada. Obesity Rev.

[38] Kirchengast S, Schober E (2006). To be an immigrant: a risk factor for developing overweight and obesity during childhood and adolescence?. J Biosocial Sci.

[39] A concise report, Alberta Centre for Active Living [Internet]. Alberta Walking Survey; [cited 2015 May 13]. Available from: http://www.albertahealthservices.ca/HealthWellness/hi-hw-al-wc-survey.pdf.

[40] Abdel-Hamid TK (2009). Thinking in circles about obesity, applying systems thinking to weight management.

[41] Raghunathan R, Naylor RW, Hoyer WD (2006). The unhealthy = tasty intuition and its effects on taste inferences, enjoyment, and choice of food products. J Am Market Ass.

[42] Hollands S, Campbell MK, Gilliland J, Sarma S (2013). A spatial analysis of the association between restaurant density and body mass index in Canadian adults. Prevent Med.

[43] Sadler RC, Gilliland JA, Arku G (2013). A food retail-based intervention on food security and consumption. Inter J Environ Res Public Health.

[44] Andreyeva T, Kelly IR, Harris JL (2011). Exposure to food advertising on television: Associations with children's fast food and soft drink consumption and obesity. Economics & Human Biology.

[45] Duncan DT, Aldstadt J, Whalen J (2011). Validation of Walkscore for estimating neighborhood walkability; an analysis of four US metropolitan areas. Inter J Environ Res. Public Health.

[46] Sandalack BA, Uribe FGA, Zanjani AE (2013). Neighbourhood type and walkshed size. J Urbanism: Inter Res Placemaking Urban Sustain.

[47] McCormack G, Giles-Corti B, Lange A (2004). An update of recent evidence of the relationship between objective and self-report measure of the physical environment and physical activity behaviours. J Sci Med Sport.

[48] Laxer RE, Janssen I (2013). The proportion of youth's physical inactivity attributable to neighborhood built environment features. Inter J Health Geog.

[49] Carr LJ, Dunsiger SI, Marcus BH (2010). Walk Score as a global estimate of neighbourhood walkability. Am J Prevent Med.

[50] Dewulf B, Neutens T, Dyck DV (2012). Correspondence between objective and perceived walking time to urban destinations: influence of physical activity, neighbourhood walkability, and socio-demographics. Inter J Health Geog.

[51] Dyck DV, Sallis JF, Cardon G (2013). Associations of neighbourhood characteristics with active park use: an observational study in two cities in the USA and Belgium. Inter J Health Geog.

[52] Kligerman M, Sallis JF, Ryan S (2007). Association of neighborhood design and recreation environment variables with physical activity and body mass index in adolescents. Am J Health Promotion.

[53] Karanfil O, Moore T, Finley P (2011). A multi-scale paradigm to design policy options for obesity prevention: exploring the integration of individual-based modeling and system dynamics.

[54] Homer J, Milstein B, Dietz W (2006). Obesity Population Dynamics: Exploring historical growth and plausible future in the US.

[55] Ahmad S, Simonovic SP (2004). Spatial system dynamics: new approach for simulation of water resources systems. J Comp Civil Eng.

[56] Fotheringham AS, Brunsdon C, Charlton M (2002). Geographically Weighted Regression: The analysis of spatially varying relationships.

[57] Mahamoud A, Roche B, Homer J (2013). Modeling the social determinants of health and simulating short-term and long-term intervention impacts for the city of Toronto, Canada. Social Sci & Med.

[58] Bailie A, Beckstead C PEMBINA Institute [Internet].

[59] Statistics Canada (2006). Community Profiles.

[60] Gauvin L, Robitaille R, Riva M (2007). Conceptualizing and operationalizing and neighbourhoods: the conundrum of identifying territorial units. Can J Public Health.

[61] Riger S, Lavrakas PJ (1981). Community ties: patterns of attachment and social interaction in urban neighborhoods. Am J Com Psycho.

[62] Grigsby-Toussaint D, Chi S-H, Fiese BH (2011). Where they live, how they play: neighborhood greenness and outdoor physical activity among preschoolers. Inter J Health Geog.

[63] Routine immunization schedule [Internet].

[64] (2013). PCTF (Postal Code Translator File).

[65] ESRI: Environmental System Research Institute [Internet].

[66] Preston S, Heuveline P, Guillot M (2000). Demography: Measuring and Modeling Population Processes.

[67] Census Dictionary (2006). Statistics Canada, Catalogue no. 92-566-X.

[68] DMTI EPOI (2011). Enhanced Point of Interest, User Manual, V2011.3.

[69] Schoeppe S, Duncan MJ, Badland H (2014). Associations between children's independent mobility and physical activity. BMC Public Health.

[70] Jones LR (2010). Investigating neighborhood walkability and its association with physical activity levels and body composition of a sample of Maryland adolescent girls. E & B T D.

[71] Walkscore [Internet].

[72] Open data, Pathways and Bikeway [Internet].

[73] Brunsdon C, Charlton M, Harris P (2012). Living with collinearity in local regression models.

[74] Cho S-H, Lambert DM, Chen Z (2010). Geographically weighted regression bandwidth selection and spatial autocorrelation: an empirical example using Chinese agriculture data. App Eco L.

[75] Wheeler D, Calder CA (2007). An assessment of coefficient accuracy in linear regression models with spatially varying coefficients. J Geog Sys.

[76] Brunsdon C, Fotheringham AS, Charlton ME (1996). Geographically weighted regression: a method for exploring spatial non-stationarity. Geog Anal.

[77] Weisent J, Rohrbach B, Dunn J, Odoi A (2012). Socioeconomic determinants of geographic disparities in campylobacteriosis risk: a comparison of global and local modeling approaches. Inter J Health Geog.

[78] Mennis J (2006). Mapping the results of geographically weighted regression. Cart J.

[79] Ingalls EJ, Kolesar P, Walker WE (2008). Using simulation to develop and validate analytic models: some case studies. Operations Res.

[80] Hovmand PS, Pitner R (2005). Combining system dynamics, social networks, and geographic information systems, perception of safety.

[81] Forrester JW (1971). Principles of Systems.

[82] Sterman JD (2000). Business Dynamics: System Thinking and Modeling for a Complex World.

[83] Vensim. Ventana Systems Inc. [Internet].

[84] Babb C, Burke M, Tranter P (2011). Developing neighbourhood ‘walkability’ indices for children's active transport, Proceedings of the Healthy City and Planning, Track 18, 3rd World Planning Schools Congress, Perth (WA.

[85] Holt NL, Spence JC, Sehn ZL, Cutumisu N (2008). Neighborhood and developmental differences in children's perceptions of opportunities for play and physical activity. Health & Place.

[86] Finn K, Johannsen N, Specker B (2003). Factors associated with physical activity in preschool children. J Pedi.

[87] Weinberger R, Sweet MN (2012). Integrating walkability into planning practice, Transportation Research Record. J Transport Res B.

[88] Robinson WS (2009). Ecological correlations and the behavior of individuals. Inter J Epidem.

[89] Openshaw S (1977). A geographical solution to scale and aggregation problems in region- building, partitioning, and spatial modelling. Transact I Brit Geog, New series.

[90] Parenteau M-P, Sawada MC (2011). The modifiable areal unit problem (MAUP) in the relationship between exposure to NO2 and respiratory health. Inter J Health Geog.

